# Rapid inactivation of SARS-CoV-2 by titanium dioxide surface coating

**DOI:** 10.12688/wellcomeopenres.16577.2

**Published:** 2021-09-09

**Authors:** Petra Micochova, Ambika Chadha, Timi Hesseloj, Franca Fraternali, Jeremy J. Ramsden, Ravindra K. Gupta

**Affiliations:** 1 Cambridge Institute of Therapeutic Immunology & Infectious Disease (CITIID), University of Cambridge, Cambridge, UK; 2 University of Cambridge Addenbrooke's Hospital Cambridge, Cambridge, UK; 3 Invisismart Technologies, London, UK; 4 Randall Centre for Cell and Molecular Biophysics, King's College London, London, UK; 5 Development and Homeostasis of the Nervous System Laboratory, The Francis Crick Institute, London, UK; 6 Clore Laboratory, University of Buckingham, Buckingham, UK

**Keywords:** Surface, SARS-CoV-2, coating, spray, TiO2

## Abstract

**Background: **Severe acute respiratory syndrome coronavirus 2 (SARS-CoV-2) transmission occurs via airborne droplets and surface contamination. Titanium dioxide (TiO _2_) coating of surfaces is a promising infection control measure, though to date has not been tested against SARS-CoV-2.

**Methods**: Virus stability was evaluated on TiO _2_- and TiO _2_–Ag (Ti:Ag atomic ratio 1:0.04)-coated 45 x 45 mm ceramic tiles. After coating the tiles were stored for 2–4 months before use. We tested the stability of both SARS-CoV-2 Spike pseudotyped virions based on a lentiviral system, as well as fully infectious SARS-CoV-2 virus. For the former, tile surfaces were inoculated with SARS-CoV-2 spike pseudotyped HIV-1 luciferase virus. At intervals virus was recovered from surfaces and target cells infected. For live virus,  after illuminating tiles for 0–300 min virus was recovered from surfaces followed by infection of Vero E6 cells. % of infected cells was determined by flow cytometry detecting SARS-CoV-2 nucleocapsid protein 24 h post-infection.

**Results: **After 1 h illumination the pseudotyped viral titre was decreased by four orders of magnitude. There was no significant difference between the TiO _2_ and TiO _2_–Ag coatings. Light alone had no significant effect on viral viability. For live SARS-CoV-2, virus was already significantly inactivated on the TiO _2_ surfaces after 20 min illumination. After 5 h no detectable active virus remained. Significantly, SARS-CoV-2 on the untreated surface was still fully infectious at 5 h post-addition of virus. Overall, tiles coated with TiO _2_ 120 days previously were able to inactivate SARS-CoV-2 under ambient indoor lighting with 87% reduction in titres at 1h and complete loss by 5h exposure.

**Conclusions**: In the context of emerging viral variants with increased transmissibility, TiO _2_ coatings could be an important tool in containing SARS-CoV-2, particularly in health care facilities where nosocomial infection rates are high.

## Background

Respiratory droplets are believed to be the major vehicle of severe acute respiratory syndrome coronavirus 2 (SARS-CoV-2) transmission. Droplets or other body fluids from infected individuals can contaminate surfaces and viable virus has been detected on such surfaces, including surgical masks, for hours, even days depending on different factors including humidity, temperature and type of surface ^1– 3^. One therefore infers that any external contamination of personal protective equipment (PPE) may last hours or even days.

Recently, there has been a further increase in SARS-CoV-2 cases globally, despite severe mitigation measures following the first wave in the first half of 2020. The new UK, Brazilian and South African variants (501Y.V1/V2/V3) have led to global anxiety and high levels of nosocomial transmission within hospitals are being observed in the 2020/21 UK winter despite universal adoption of wearing face masks, regular testing of staff and patients, and social distancing measures. It may well be that contamination of surfaces is now disproportionately contributing to transmission ^4, 5^.

Traditional forms of decontamination (such as alcohol-based sprays, quaternary ammonium compounds, and sodium hypochlorite and other chlorine-based compounds) require repeated applications. Photocatalytic surfaces, on the other hand, permanently oxidize, inactivate and destroy microorganisms under normal ambient lighting conditions ^6^. A recent hospital study of titanium dioxide-coated surfaces demonstrated progressive lowering of the bacterial bioburden ^7^. Moreover, the radicals are not considered to induce antimicrobial resistance ^8^. TiO _2_ is especially attractive because it is considered nontoxic to humans: titanium, coated with its oxide, is the most widely used material for implants ^9^. TiO _2_ is also exceedingly stable, unlike other photocatalysts such as zinc oxide and tungsten trioxide. Illumination of TiO _2_ generates highly oxidizing free radicals that are known to have bactericidal and antiviral action against influenza and rotavirus ^10– 12^. SARS-CoV-2 has not hitherto been investigated.

## Methods

### Cell lines

293T and Vero E6 cells were cultured in DMEM complete (DMEM, Sigma D5030) supplemented with 100 U/ml penicillin (Sigma), 0.1 mg/ml streptomycin (Sigma), and 10% fetal calf serum, GIBCO, Thermofisher). Vero E6 were a gift from Prof. Ian Goodfellow. 293T cells were a gift from Prof Greg Towers. ACE-2/TMPRSS2-expressing 293T cells were generated by transfecting plasmids expressing ACE-2/TMPRSS2 from a CMV promoter in pCDNA3.1 (Thermofisher Cat no: V79020) ^13^. 

### Pseudotyped virus

SARS-CoV-2 Spike pseudotyped HIV-1 luciferase particles were produced by transfection of 293T cells with 1ug pCAGGS-SARS-CoV-2 Spike expressing plasmid (NIBSC cat no: 100976), 1ug p8.91HIV-1 gag-pol expression plasmid (a gift from Prof Greg Towers) and 1.5ug pCSFLW (expressing the firefly luciferase reporter gene with the HIV-1 packaging signal – a gift from Prof Greg Towers) ^14^. Plasmids were mixed in Optimem (GIBCO, Thermofisher Cat no: 31985062)) Following transfection in 10cm plastic petri dishes (Nunc cat no:150464), viral supernatant was collected at 48 and 72 h after transfection, filtered through a 0.45 μm filter (Millipore, cat no: HAWP04700) and stored at −80 °C. The 50% tissue culture infectious dose (TCID _50_) of SARS-CoV-2 pseudovirus was determined using the Steady-Glo luciferase (Promega cat no: E2550) assay system including a luminometer (Glomax Navigator Luminometer, Promega, cat no: GM2000).

### Viral isolate

Live SARS-CoV-2 (SARS-CoV-2/human/Liverpool/REMRQ0001/2020) used in this study was isolated by Lance Turtle (University of Liverpool), David Matthews and Andrew Davidson (University of Bristol). A SARS-CoV-2 virus stock was produced by infecting Vero E6 cells at MOI 0.01. Culture supernatant was collected 48 h post-infection. The titre of the stock was determined by adding tenfold serial dilutions of virus onto Vero E6 cells. 24 h post-infection cells were fixed by removing media and replacing with 3% paraformaldehyde in PBS. Samples were stained for nucleocapsid protein using a monoclonal rabbit anti-Nucleocapsid antibody (1:1000, MA5-36086, ThermoFisher) and % infection determined by flow cytometry on a BD FACSCalibur instrument, with 10,000 cells were counted. SARS-CoV-2 virus titres were determined as infectious units per ml (IU/ml) as follows: (% infected cells) × (total number of cells) × (dilution factor) / volume of inoculum added to cells.

### Surfaces and illumination

Ceramic tiles were wiped down with neutral disinfectant then coated with either a TiO2 based solution or with a combination of TiO2 and Ag using a spray gun (bespoke). The tiles were allowed to dry for 15 minutes. The spray gun was connected to an 11L air compressor (Makita). The nozzle orifice was 8 microns with pressure fixed at 12 PSI to atomise the coating. Virus stability was evaluated on the following surfaces: sterile untreated Sterilin standard Petri dish; TiO _2_- and TiO _2_–Ag (Ti:Ag atomic ratio 1:0.04)-coated 45 × 45 mm ceramic tiles (Invisi Smart Technologies UK Ltd). The coatings are transparent and colourless and therefore invisible to the human eye. After coating the tiles were stored for 2–4 months before use. Surfaces were exposed (610 lx, ambient laboratory light) for 1 h before the start of each experiment to ensure a steady state of radical generation. The same light was used during virus exposure, during which relative humidity was approximately 65% and temperature 21 °C (in a microbiological safety cabinet).

### Surface inoculation and sampling

***SARS-CoV-2 spike pseudotyped virus inactivation.*** Tile surfaces were inoculated with 10 ^5^ RLU of SARS-CoV-2 spike pseudotyped HIV-1 luciferase virus onto the surface of the tiles at a dosage of 5 μl over 5 x 5 mm at time *t* = 0 and illuminated for up to 6 h. Three individual 5x5mm spots were used on the same tile. At intervals virus was recovered from surfaces with 50uL of DMEM complete followed by infection of ACE-2/TMPRSS2-expressing 293T cells. Luminescence was measured using Steady-Glo Luciferase assay system (Promega) 48 h post-infection.

***SARS-CoV-2 live virus inactivation.*** 6×10 ^6^ IU/ml of SARS-CoV-2 virus was added onto the surface of the tiles at a dosage of 2 μl over 5 × 5 mm. Three individual 5x5mm spots were used on the same tile. After illuminating for 0–300 min virus was recovered from surfaces with 50uL of DMEM complete followed by infection of Vero E6 cells. % of infected cells was determined by flow cytometry detecting SARS-CoV-2 nucleocapsid protein 24 h post-infection.

### Kinetic analysis of inactivation

The main challenge is that laboratory inactivation experiments are necessarily carried out with large numbers of viruses, with which the inactivating material is brought into contact at the beginning of the experiment, and the decay of the entire virus population is measured ^15^. What is of practical interest in the scenario of a coating designed to keep surfaces (e.g., in a hospital) free of viral (and bacterial) bioburden is how quickly an individual virus is inactivated. According to analysis of previously reported results for influenza virus inactivation ^11^, the kinetics fit a convective diffusion transport model even in the absence of mechanical agitation, most likely due to almost inevitable thermal gradients ^15^. The concentration of survivors is thereby predicted to follow a so-called exponential decrease, and plotting the logarithm of the number of survivors v. time should give a straight line, the slope of which is – *k*, the inactivation rate coefficient. The value of *k* can then be compared with the transport-limited fastest possible rate calculated from the size of the virus ^15^. 

### Statistical analysis

We did not perform statistical analyses in this work.

## Results

**Figure 1.**
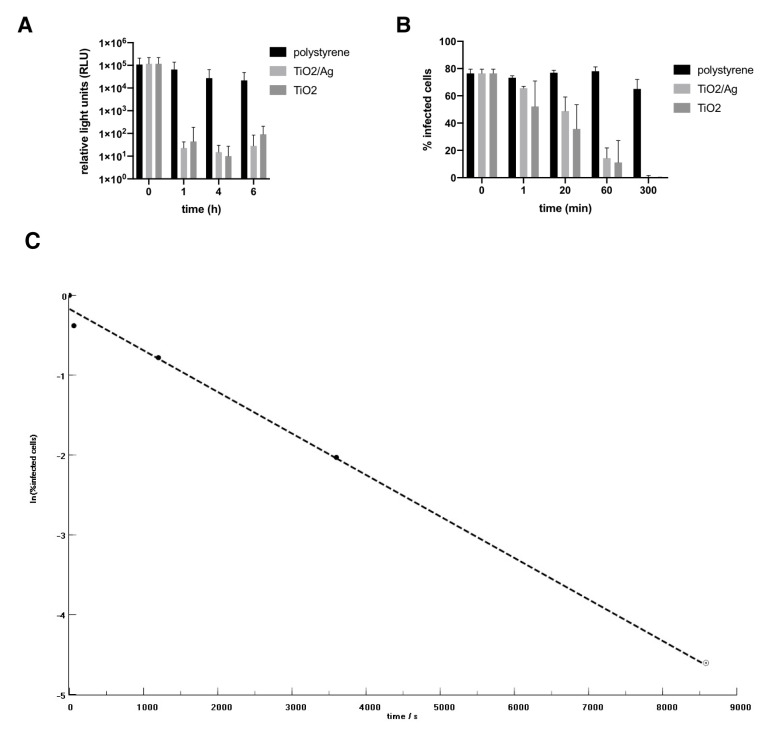
Effect of Ti0 _2 _/Ag and Ti0 _2 _coated tiles on. ( **A**) SARS-CoV-2 Spike pseudotype viral infection and ( **B**) SARS-CoV-2 isolate REMRQ0001/Human/2020/Liverpool ( **C**) Plot of the experimental data from ( **B**) as ln(%infected cells) v. time (s). Dashed line represents a linear regression using the first 4 data points. The slope of the line yields a disinfection rate coefficient *k*.

After 1 h illumination the pseudotyped viral titre was decreased by four orders of magnitude ( Figure 1A ^16^). There was no significant difference between the TiO _2_ and TiO _2_–Ag coatings. Light alone had no significant effect on viral viability.

Next, we tested the ability of the coated tiles to inhibit fully infectious live virus. Coated and uncoated surfaces were exposed to SARS-CoV-2. Virus was harvested at the times indicated and used to infect Vero E6 target cells. SARS-CoV-2 was already significantly inactivated on the TiO _2_ surfaces after 20 min illumination. After 5 h no detectable active virus remained ( Figure 1B ^16^). Significantly, SARS-CoV-2 on the untreated surface was still fully infectious at 5 h post-addition of virus. TiO _2_–Ag appeared somewhat less effective than TiO _2_ alone, but the difference was not significant.

Plotting the experimental data ( Figure 1B ^16^) as ln(titre) v. time ( Figure 1C ^16^) yields a disinfection rate coefficient *k* of (5.2 ± 0.6) × 10 ^–4^ s ^–l^, which corresponds to the transport-limited fastest possible rate estimated for SARS-CoV-2 approaching a disinfecting surface in water ^15^. Hence we infer that the viruses arriving at the surface from the inoculum are essentially immediately inactivated. From our illumination conditions we estimate the generation rate of radicals as about 10 ^13^ cm ^–2^ s ^–l^, ^6^ corresponding to about 800 radicals s ^–l ^over the area occupied by one virus at the surface, By extrapolating the data from the first four points to the assumed detection limit, it can be seen that very likely no detectable virus from the initial inoculum remained soon after 2 h exposure ( Figure 1C ^16^).

## Discussion

The potent extended anti- SARS-CoV-2 effect of titanium dioxide surface coatings is highly desirable in hospital settings where both patients and staff might be shedding viruses. An important advantage of these surfaces is that they can be activated by ordinary interior light and do not need UV irradiation, which is usually incompatible with simultaneous human presence. The coating has a rough surface with high local curvature that creates an absorption tail into the blue region of the visible spectrum ^6^, overlapping the spectral output of ordinary interior lighting. This is sufficient to ensure an adequate rate of radical generation for effectively immediately inactivating viruses and other microorganisms arriving from the air or hand touches. Conversely, a limitation is that if a sudden very large contamination event occurred, particularly one that severely diminished the light reaching the photocatalyst, it might take impracticably long for the contamination to be eliminated. Hence, in that case rough cleaning, even washing with water, should be used to remove the gross contamination. Further limitations to our study include the lack of a control tile that was unsprayed for the virus isolation work, and the fact that activity against droplets from infected individuals may be different as a result of other material such as mucus present. Controlled experiments with respiratory secretions would not be feasible.

The efficacy of the TiO _2_ coating under typical hospital lighting makes it a promising candidate for enhancing the protection afforded by facemasks and other PPE, and well as surfaces likely to be contaminated and hence acting as reservoirs for transmitting infection if left untreated. The coatings would need only periodic re-application depending on the amount of wear, for example 6-12 monthly, and would be widely available in the future. Such interventions are increasingly critical in conditions where viral variants with increased transmissibility are the new norm ^14, 17– 19^.

## Data availability

### Underlying data

DRYAD: SARS-CoV-2 viability after exposure to titanium dioxide coated tiles. https://doi.org/10.5061/dryad.4j0zpc89z ^16^

This project contains the following underlying data:

- PM_luminometer_data-1.xlsx (Luminometer data for SARS-CoV-2 spike pseudotyped virus inactivation)
- TiO2_fcs_files.zip (Flow cytometry data for SARS-CoV-2 live virus inactivation)

Data are available under the terms of the Creative Commons Zero "No rights reserved" data waiver (CC0 1.0 Public domain dedication).
