# Supplementary material for: Rapid inactivation of SARS-CoV-2 by titanium dioxide surface coating
Source: Wellcome Open Res. 2021 Sep 9;6:56. Originally published 2021 Mar 11. [Version 2] doi: 10.12688/wellcomeopenres.16577.2 (PMC8450774; doi:10.12688/wellcomeopenres.16577.2)
Supplement: Peer review history

# Peer review: Reviewer response for version 2

**DOI:** 10.21956/wellcomeopenres.19014.r45807

**Published:** 2021-09-17

**Authors:** Gert U. van Zyl

**Affiliations:** 1 National Health Laboratory Service, Tygerberg Hospital, Cape Town, South Africa; 2 Division of Medical Virology, Stellenbosch University, Stellenbosch, South Africa

The authors have adequately addressed all my minor concerns.

Is the work clearly and accurately presented and does it cite the current literature?

Yes

If applicable, is the statistical analysis and its interpretation appropriate?

Not applicable

Are all the source data underlying the results available to ensure full reproducibility?

Partly

Is the study design appropriate and is the work technically sound?

Yes

Are the conclusions drawn adequately supported by the results?

Yes

Are sufficient details of methods and analysis provided to allow replication by others?

Partly

Reviewer Expertise:

Diagnostic and clinical virology, HIV persistence, HIV drug resistance, viral epidemiology

I confirm that I have read this submission and believe that I have an appropriate level of expertise to confirm that it is of an acceptable scientific standard.

# Peer review: Reviewer response for version 2

**DOI:** 10.21956/wellcomeopenres.19014.r45808

**Published:** 2021-09-13

**Authors:** Xuan Xue

**Affiliations:** 1 School of Pharmacy, University of Nottingham, Nottingham, UK

I have no further comments to make.

Is the work clearly and accurately presented and does it cite the current literature?

Partly

If applicable, is the statistical analysis and its interpretation appropriate?

Not applicable

Are all the source data underlying the results available to ensure full reproducibility?

Partly

Is the study design appropriate and is the work technically sound?

Yes

Are the conclusions drawn adequately supported by the results?

Yes

Are sufficient details of methods and analysis provided to allow replication by others?

Partly

Reviewer Expertise:

Antiviral materials, surface chemistry, polymer chemistry, biomaterials, high throughput screening

I confirm that I have read this submission and believe that I have an appropriate level of expertise to confirm that it is of an acceptable scientific standard.

# Peer review: Reviewer response for version 1

**DOI:** 10.21956/wellcomeopenres.18269.r45067

**Published:** 2021-07-26

**Authors:** Xuan Xue

**Affiliations:** 1 School of Pharmacy, University of Nottingham, Nottingham, UK

In this research article, the authors demonstrated that titanium dioxide (TiO _2_)- and TiO _2_-Ag coating of surfaces can be a promising measure to reduce the fomite transmission of SARS-CoV-2 for future infection protection control. TiO _2 _has been previously demonstrated to have bactericidal and antiviral action against influenza and rotavirus, but not yet studied on SARS-CoV-2, by generating oxidizing free radicals under light. This research compared the stability of SARS-CoV-2 Spike pseudo virus and infectious SARS-CoV-2 virus on coated and uncoated surfaces, and the results of which evidenced that TiO _2_- and TiO _2_-Ag coatings, compared to the uncoated control surfaces, effectively inactivated both SARS-CoV-2 pseudo and live viruses under ambient indoor light within hours. The authors indicated the potential of TiO _2_ coatings as a tool in contribution to the control of SARS-CoV-2 transmission in public and health care settings. In this manuscript, the work has been well designed, clearly presented and concluded with relative adequate data.

 Some minor comments are stated below:

1. There are a few different types of materials, e.g. metal-based, silicon-based, carbon-based, and polymers, that have been showing antiviral potentials. It would be good to briefly review the current literature on them and indicate the merit for investigating more on TiO _2_- coatings than others.
2. The coating procedure was described in the methods section without further optimisation. Also, there are no further discussions on the validation and evaluation of the successful coatings on the surface. A series of surface characterisation techniques are therefore recommended, for example, ToF-SIMS and XPS to confirm the surface chemistries and their distributions after coating, and SEM to show the surface features and uniformity of the coating.
3. The virus stability was evaluated on both TiO _2_- coated and uncoated (control) surfaces. According to Fig. 1 B, polystyrene surface was used as the control substrate. However, as the coatings were applied on tiles (please specify its material) and no data have evidenced the successful coating (please see Comment 2), an uncoated tile surface would be a more appropriate control substrate compared to polystyrene.
4. In order to recommend on effective inactivation of virus on TiO _2_- coated surfaces for health care facilities in the future, it would be good to have more details of the light applied in this research as well as investigate more on different lights, in terms of their type, power, and applied distance to surfaces, in this study.  

Is the work clearly and accurately presented and does it cite the current literature?

Partly

If applicable, is the statistical analysis and its interpretation appropriate?

Not applicable

Are all the source data underlying the results available to ensure full reproducibility?

Partly

Is the study design appropriate and is the work technically sound?

Yes

Are the conclusions drawn adequately supported by the results?

Yes

Are sufficient details of methods and analysis provided to allow replication by others?

Partly

Reviewer Expertise:

Antiviral materials, surface chemistry, polymer chemistry, biomaterials, high throughput screening

I confirm that I have read this submission and believe that I have an appropriate level of expertise to confirm that it is of an acceptable scientific standard, however I have significant reservations, as outlined above.

# Response

**Published:** 2021-09-07

**Authors:** Ravindra Gupta

Reviewer 2:

 In this research article, the authors demonstrated that titanium dioxide (TiO _2_)- and TiO _2_-Ag coating of surfaces can be a promising measure to reduce the fomite transmission of SARS-CoV-2 for future infection protection control. TiO _2 _has been previously demonstrated to have bactericidal and antiviral action against influenza and rotavirus, but not yet studied on SARS-CoV-2, by generating oxidizing free radicals under light. This research compared the stability of SARS-CoV-2 Spike pseudo virus and infectious SARS-CoV-2 virus on coated and uncoated surfaces, and the results of which evidenced that TiO _2_- and TiO _2_-Ag coatings, compared to the uncoated control surfaces, effectively inactivated both SARS-CoV-2 pseudo and live viruses under ambient indoor light within hours. The authors indicated the potential of TiO _2_coatings as a tool in contribution to the control of SARS-CoV-2 transmission in public and health care settings. In this manuscript, the work has been well designed, clearly presented and concluded with relative adequate data.

 Some minor comments are stated below:

1. There are a few different types of materials, e.g. metal-based, silicon-based, carbon-based, and polymers, that have been showing antiviral potentials. It would be good to briefly review the current literature on them and indicate the merit for investigating more on TiO _2_- coatings than others.

Response: A formal review of literature would be lengthy. Hence we have focused on the tile based Ti02 coating.

  

1. The coating procedure was described in the methods section without further optimisation. Also, there are no further discussions on the validation and evaluation of the successful coatings on the surface. A series of surface characterisation techniques are therefore recommended, for example, ToF-SIMS and XPS to confirm the surface chemistries and their distributions after coating, and SEM to show the surface features and uniformity of the coating.

Response: Unfortunately these experiments are beyond the scope of this report but are important as the reviewer points out.

# Peer review: Reviewer response for version 1

**DOI:** 10.21956/wellcomeopenres.18269.r43031

**Published:** 2021-03-17

**Authors:** Gert U. van Zyl

**Affiliations:** 1 National Health Laboratory Service, Tygerberg Hospital, Cape Town, South Africa; 2 Division of Medical Virology, Stellenbosch University, Stellenbosch, South Africa

Micochova *et al.* described the inactivation of SAR-CoV-2 on surfaces coated with titanium dioxide (TiO _2_). TiO _2 _is photocatalytic under ambient light, producing oxidative radicals that inactivate infectious agents. The inactivation of SARS-CoV-2 pseudotyped virus and live SARS-CoV-2 cultures were assessed on ceramic tiles coated with either TiO _2 _or TiO _2_-silver(Ag) by culturing viruses from these surface at different time intervals. The authors found that after 5 hours, no infective virus remained on the treated surface but it was still fully infectious on the untreated surface.

 The manuscript adds valuable novel data and provides an apparently practicable solution to coating surfaces in hospitals, which would result in surface disinfection under ambient light. The manuscript is overall well-constructed, accessible and well-written. 

**I have a few minor comments for improvement of overall clarity:**

1. Please clarify how the photocatalytic activity of TiO _2 _is affected by natural vs incandescent vs fluorescent light bulbs?
2. Please describe the time intervals and procedure of sampling to assess infectivity in detail (were different spots sampled on the same surface?)
3. Please clarify the nature of the control surface used. The text referred to an “uncoated surface” which I assumed would be ceramic, but the figure refers to polystyrene (is the latter an appropriate control surface?)
4. Considering the potential of this surface coating: Please clarify a) how frequently the coating would have to be reapplied, b) what the cost of coating per square meter would be and c) how widely available TiO _2 _is for use as a spray for surface coating.
5. Please comment on the difference of respiratory droplets that contain mucous vs free cultured virus and how this may have affected the inactivation of viral particles.

Is the work clearly and accurately presented and does it cite the current literature?

Yes

If applicable, is the statistical analysis and its interpretation appropriate?

Not applicable

Are all the source data underlying the results available to ensure full reproducibility?

Partly

Is the study design appropriate and is the work technically sound?

Yes

Are the conclusions drawn adequately supported by the results?

Yes

Are sufficient details of methods and analysis provided to allow replication by others?

Partly

Reviewer Expertise:

Diagnostic and clinical virology, HIV persistence, HIV drug resistance, viral epidemiology

I confirm that I have read this submission and believe that I have an appropriate level of expertise to confirm that it is of an acceptable scientific standard, however I have significant reservations, as outlined above.

# Response

**Published:** 2021-09-07

**Authors:** Ravindra Gupta

Reviewer 1:

 Micochova *et al.* described the inactivation of SAR-CoV-2 on surfaces coated with titanium dioxide (TiO _2_). TiO _2 _is photocatalytic under ambient light, producing oxidative radicals that inactivate infectious agents. The inactivation of SARS-CoV-2 pseudotyped virus and live SARS-CoV-2 cultures were assessed on ceramic tiles coated with either TiO _2 _or TiO _2_-silver(Ag) by culturing viruses from these surface at different time intervals. The authors found that after 5 hours, no infective virus remained on the treated surface but it was still fully infectious on the untreated surface.

 The manuscript adds valuable novel data and provides an apparently practicable solution to coating surfaces in hospitals, which would result in surface disinfection under ambient light. The manuscript is overall well-constructed, accessible and well-written. 

**I have a few minor comments for improvement of overall clarity:**

1. Please clarify how the photocatalytic activity of TiO _2 _is affected by natural vs incandescent vs fluorescent light bulbs?

Response: Both natural (i.e. sunlight) and incandescent filament lamps approximate to black body radiators, albeit with different temperatures. The photocatalytic activity, which depends on the convolution of the incident light with the absorption spectrum of the TiO2, is therefore qualitatively the same for the two. The degree of activity (as measured e.g. by rate of inactivation of viruses) will simply depend on the actual irradiances -- to a first approximation linearly. Fluorescent lighting has a different spectral distribution. For a good estimate of the degree of photocatalytic activity, one can simply take the irradiance in the near UV-violet-blue spectral range and compare it with that of the natural or incandescent source.

1. Please describe the time intervals and procedure of sampling to assess infectivity in detail (were different spots sampled on the same surface?)
  This has now been described in the methods in more detail

 

1. Please clarify the nature of the control surface used. The text referred to an “uncoated surface” which I assumed would be ceramic, but the figure refers to polystyrene (is the latter an appropriate control surface?)

Response: We used plastic as a control surface in the absence of an uncoated tile. We have now stated this in the limitations section.

  

1. Considering the potential of this surface coating: Please clarify a) how frequently the coating would have to be reapplied, b) what the cost of coating per square meter would be and c) how widely available TiO _2 _is for use as a spray for surface coating.

Response: We have now specified in the paper that the coating would be re-applied every 6-12 months.  The frequency depends on the adhesion of the coating and the degree of wear to which it is subjected. With no wear the coating lasts indefinitely. With moderate hand-touching the coatings used in this work will typically last 6-12 months

 The cost is a few dollars per sq m and the coating is planned to be made widely available. The last two comments cannot really be put in the text as this is a changing area

  

1. Please comment on the difference of respiratory droplets that contain mucous vs free cultured virus and how this may have affected the inactivation of viral particles.

Response: Mucous mainly contains mucin, a glycoprotein family, which would be photocatalytically oxidized to CO2 and H2O by TiO2. Partially oxidized intermediates may themselves be radicals that would damage and inactivate the viruses. Without detailed mechanistic investigations it is difficult to predict whether the mucous accelerates or retards virus inactivation. The ultimate outcome is not expected to be different, however. We have now commented on this in the discussion and thank the reviewer for this point.
